# Profil épidémiologique et anatomopathologique du cancer colorectal: à propos de 36 caswe

**DOI:** 10.11604/pamj.2018.30.159.15061

**Published:** 2018-06-22

**Authors:** Mohamed Said Belhamidi, Mohamed Sinaa, Abdessamad Kaoukabi, Hicham Krimou, Mohamed Menfaa, Farid Sakit, Abdelkrim Choho

**Affiliations:** 1 Service de Chirurgie Viscérale, Hôpital Militaire Moulay Ismail, Meknès, Maroc; 2 Laboratoire d’Anatomopathologie, Hôpital Militaire Moulay Ismail, Meknès, Maroc

**Keywords:** Cancer colorectal, anatomopathologie, adénocarcinome, Colorectal cancer, anatomopathology, adenocarcinoma

## Abstract

Le cancer colorectal est classé parmi les cancers les plus fréquents au monde (3^ème^ rang après le cancer du sein et celui de la prostate), deuxième cancer digestif au Maroc après celui de l'estomac. Cependant son incidence dans notre pays reste moins élevée que celle des pays occidentaux (2.5 à 3.3/100 000 ha) mais rejoint celle des pays du Maghreb dont ce cancer touche les sujets jeunes dans 27% des cas. Le cancer colorectal (CCR) est un des meilleurs exemples du processus multi étape de cancérogenèse. La connaissance des caractéristiques anatomopathologique du CCR conditionnera certainement nos attitudes thérapeutiques. Nous avons mené une étude rétrospective épidémiologique et anatomopathologique au service de chirurgie viscérale de l'hôpital militaire Moulay Ismail à Meknès sur une période de 5 ans allant du mois de janvier 2012 au mois de Décembre 2016 (36 cas: 14 femmes et 22 hommes). À la lumière de cette étude, l'objectif de ce travail est d'analyser le profil épidémiologique et les aspects anatomopathologiques des cancers colorectaux. L'analyse de nos résultats montre un profil épidémiologique particulier qui se caractérise par un âge plus jeune, une répartition selon le sexe en faveur des hommes, les carcinomes sporadiques sont largement prédominants avec une répartition topographique recto sigmoïdienne fréquemment observée. Sur le plan pronostique, le faible degré de différenciation des adénocarcinomes et le type mucineux sont corrélés à un stade TNM et d'Aster Coller avancés, avec un statut ganglionnaire péjoratif. Cette approche multidisciplinaire sera une première nationale, rendant ainsi notre structure de pratique et de recherche médicale l'une des plateformes de prise en charge multidisciplinaire du cancer colorectal au Maroc.

## Abstract (en)

Colorectal cancer is classified among the most common cancers in the world (after breast cancer and prostate cancer) and it is the second digestive tract cancer in Morocco after stomach cancer. However, its incidence in our country is lower than that of western countries (2.5 to 3.3 / 100 000 ha) but it coincides with that of Maghreb countries where this cancer affects young subjects in 27% of cases. Colorectal cancer (CRC) is one of the best examples of multistep carcinogenesis. Knowing the anatomopathological characteristics of CRC will certainly affect our therapeutic approaches. We conducted a retrospective epidemiological and anatomopathological study in the Department of Visceral Surgery at the Military Hospital Moulay Ismail, Meknes over a period of 5 years from January 2012 to December 2016. The study involved 36 patients: 14 women and 22 men. The aim of this study was to analyze the epidemiological profile and the anatomopathological features of colorectal cancers. The analysis of our results shows a specific epidemiological profile which is characteristic of a type of colorectal cancer affecting younger subjects, mainly male patients. Sporadic carcers are largely predominant, occurring mainly in the rectosigmoid region. A low degree of differentiation of adenocarcinomas with mucinous features is correlated with advanced TNM and Aster Coller staging and with lymph node status with poor prognosis. This multidisciplinary approach will be a novelty at national level, thus making our structure of clinical practice and research one of the centres for multidisciplinary management of colorectal cancer in Morocco.

## Introduction

Le cancer colorectal est, par sa fréquence et sa gravité, un problème important de santé publique dans les pays développés à population vieillissante. Le cancer colorectal occupe le second rang des affections malignes en terme d'incidence et de mortalité dans les pays riches. Il est plus rare en Amérique du Sud, en Asie à l'exception du Japon et encore plus rare en Afrique noire. La plupart des cancers colorectaux apparaissent après l'âge de 60 ans. Ils surviennent le plus souvent de manière sporadique et il ne s'agit d'une affection héréditaire que dans environ 5% des cas. L'adénocarcinome est le type histologique le plus fréquent. Les cancers colorectaux se développent le plus souvent sur des adénomes préexistants, après une période de latence de plusieurs années, ce qui les rend accessibles au dépistage et la prévention secondaire. L'alimentation est le facteur exogène de loin le plus important identifié dans l'étiologie du cancer colorectal. Le développement des techniques de biologie moléculaire a permis de mieux comprendre la genèse de ce cancer, ouvrant de nouveaux horizons à la recherche thérapeutique en quête de nouvelles molécules toujours plus efficaces. Nous rapportons une étude rétrospective portant sur 36 cas colligés au sein du service de chirurgie viscérale de l'hôpital militaire Moulay Ismail de Meknès. Le but de notre étude est d'évaluer le profil épidémiologique et anatomopathologique du cancer colorectal.

## Méthodes

Il s'agit d'une étude rétrospective descriptive menée au service de chirurgie viscérale de l'hôpital militaire Moulay Ismail de Meknès et portant sur 36 patients porteurs de cancer colorectal sur une période de 5 ans allant du mois de Janvier 2012 au mois de Décembre 2016. Les sources de données auxquelles on a eu recours étaient: les dossiers médicaux, les registres d'hospitalisation, les comptes rendus opératoires, et les registres des comptes rendus anatomo-pathologiques. Pour chaque patient, les paramètres suivants ont été révélés: nom et prénom, sexe, l'âge, origine, ATCD médicaux, familiaux, chirurgicaux, les habitudes toxiques, caractéristique de la symptomatologie, le siège tumoral, le stade, le type histologique, présence d'adénopathies, emboles vasculaire, engainements peri-nerveux et limites d'exérèse. Critères d'inclusion étaient tous les patients pris en charge dans l'HMMI et présentant un cancer colorectal de type adénocarcinome à l'histologie quel que soit son stade au moment du diagnostic.

## Résultats

**Figure 1:**
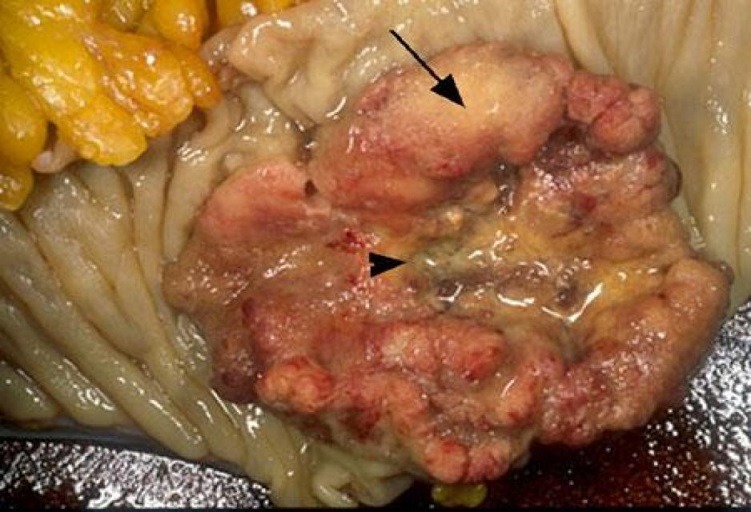
Adénocarcinome ulcéro-bourgeonnant

**Figure 2:**
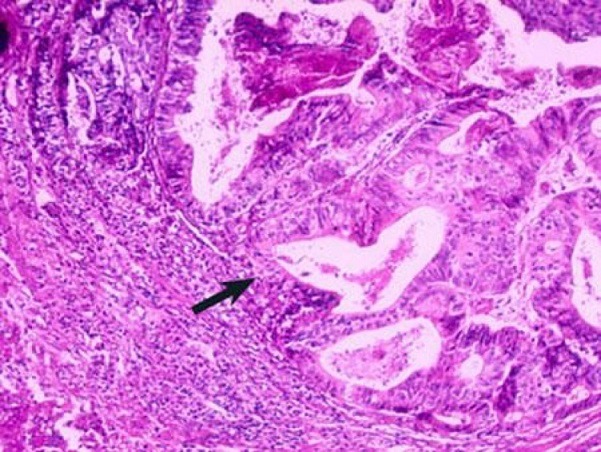
Adénocarcinome colique (grossissement moyen): la muqueuse et la sous- muqueuse sont détruites par la prolifération adénocarcinomateuse constituée de tubes irréguliers (flèche)

**Figure 3:**
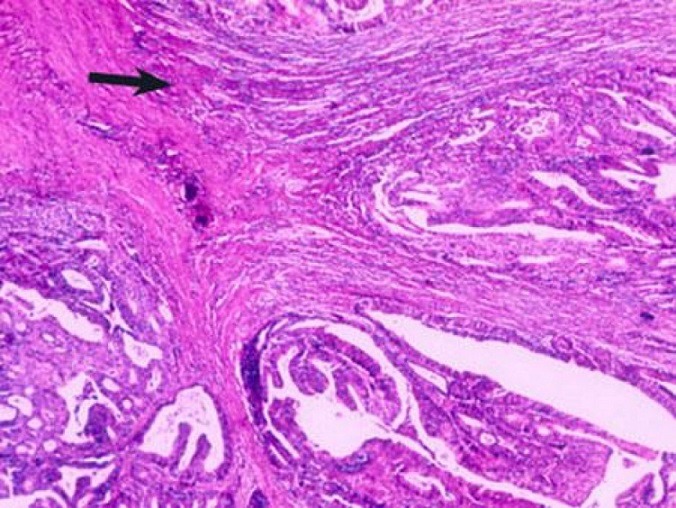
Adénocarcinome colique (grossissement moyen): envahissement de la muqueuse (flèche)

Parmi les 36 cas étudiés, il y avait 22 hommes (61%) et 14 femmes (39%) avec un sexe ratio de 1,5. La tranche d'âge varie entre 36 ans et 85 ans avec un âge moyen de 56,8 ans avec un pic entre 50 et 60 ans (45% des cas) (Figure 1). Pour l'origine géographique de nos malades, on note une prédominance de l'origine rural, 60% du milieu rural et 40% du milieu urbain. La localisation recto-sigmoidienne était la plus fréquente dans notre série (50% des cas), 6 cas (16,60%) pour le colon gauche et le même nombre de cas pour le colon droit et le colon transverse. Sur le plan macroscopique, 91,6% des tumeurs étaient sous forme ulcéro-bourgeonnante (Figure 2). Tous nos patients ont une preuve histologique de malignité et dans la totalité des cas, il s'agit d'un adénocarcinome liberkhunien, soit 97% des cas dont 58,7% étaient bien différenciés, 28% moyennement différenciés et seulement 5,3% peu différenciés (Figure 3). Pour la stadification TNM: 20 cas ont des tumeurs classées pT2 soit (55,55%), 8 cas pT3, 5 cas pT1, 3 cas pT4. La majorité des cas n'avait pas d'envahissement ganglionnaire (75% des cas). Les embols vasculaires étaient retrouvés chez 5 cas soit 13,88%. L'engainement peri-nerveux était présent chez 5 malades. Les limites d'exérèse étaient envahies chez 31 malades soit 86,11%.

## Discussion

**La fréquence et l'incidence:** Le cancer du rectum a connu une augmentation d'incidence durant ces dernières décades, avec un taux variable selon les pays. Le nombre de nouveaux cas de cancer colorectal a rapidement augmenté au niveau mondial depuis l'année 1975 [1]. C'est l'un des cancers les plus répondus dans les pays développés. Plus de 33000 nouveaux cas sont enregistrés par an dans en France [2]. En outre le cancer rectal est une maladie courante dans les Pays-Bas avec environ 4.000 nouveaux cas et 2.000 cas de décès par an [3]. Il est la deuxième cause de décès par cancer aux États-Unis [4]. Au Maroc, l'OMS estime à 1271 le nombre de nouveaux cas de cancer colorectal et à 1185 le nombre annuel de décès [5]. Il faut noter que ce ne sont que des estimations et qu'il n'existe pas de registres de cancer dans notre pays. Une étude faite au CHU Mohammed VI de Marrakech entre 2003 et 2006 a objectivé: 1) au service de gastro-entérologie 61 cas de cancer colorectal, dont 52,4% sont des cancers du rectum; 2) au service d'oncologie de Marrakech 143 cas de cancers colorectaux, dont 50,3% sont des cancers du rectum [6]. Selon une étude rétrospective s'étalant sur une période de 3 ans entre 2009 et 2011 au centre d'oncologie Hassan II d'Oujda, 100 cas de CCR ont été pris en charge (35 cas de cancer du côlon et 65 cas de cancer du rectum) [7]. Dans notre étude nous avons colliges 36 patients atteint du cancer colorectal sur 5 ans.

**L'âge:** Le cancer colorectal est rare avant 45 ans [8]. Son incidence augmente avec l'âge. L'âge moyen au diagnostic est de 69,5 pour les hommes et de 72,8 ans pour les femmes. L'incidence est identique pour les 2 sexes jusqu'à 60 ans, puis le cancer devient prédominant chez les hommes. Le ratio d'incidence entre les deux sexes augmente régulièrement entre 55 ans et 75 ans, passant de 1,0 à 1,7 [9]. Dans notre série, le sexe ratio était de 1,5. Concernant le sujet jeune, l'âge de 45 ans est considéré par la plupart des auteurs, comme la “frontière” définissant la population “jeune” chez laquelle ce cancer est rare. L'étude des incidences de ce cancer chez le sujet jeune révèle qu'il y a une forte incidence en Arabie Saoudite avec 21 à 23% des cas, l'Italie avec 17%, Le Japon avec 10%. Dans les pays occidentaux cette fréquence est faible [10-12]. Au Maroc, L'âge moyen de survenue de cancer colorectal d'après les estimations de l'institut national d'oncologie (INO) est 51, 5 ans avec 26,6 % des patients âgés de moins de 40 ans pour le cancer du rectum [13]. Dans notre série, la tranche d'âge varie entre 36 ans et 85 ans avec un âge moyen de 56,8 ans et 10% des cas avaient mois de 40 ans, ce qui est conforme aux données de la littérature.

**L'anatomopathologie:** Le tiers inférieur du rectum est le siège le plus fréquent. Dans la série de Kelli et al 52,3% des tumeurs siègent au niveau du bas rectum. Dans notre série, 50% des cas ont des tumeurs du recto-sigmoide. Pour la classification TNM, les stades T2 et T3 sont les plus fréquent dans la littérature. Dans la série de kelli, 54% des malades avaient un stade T3 et dans la série de Lahmidani et al, le stade T2 représente 61,8% [7]. Sur le plan macroscopique, la forme ulcéro-bourgeonnante est la plus fréquente. Elle représente 65% des cas alors que la forme végétante représente 25%. En comparant aux autres séries, on constate une fréquence plus élevée de la forme ulcéro-bourgeonnante dans notre étude avec une fréquence de 91,6%. Sur le plan microscopique, l'adénocarcinome liburkhunien représente 95% des cas dans la littérature [14], et la forme bien différenciée est la plus fréquente dans la plupart des séries [15-17]. Les résultats trouvés dans notre série se rapprochent de ceux rapportés dans la littérature: l'adénocarcinome bien différencié représente 58,7% des cas (Tableau 1). Dans une étude des facteurs prédictifs de récidive des cancers colorectaux avec instabilité microsatellitaire, des emboles vasculaires ont été retrouvées chez 26% des cas, des engainements péri-nerveux dans 20% des cas. Ceci concorde avec notre étude dans laquelle les emboles vasculaires et les engainements péri-nerveux étaient présents dans 13,88 des cas [18,19].

**Tableau 1:** Comparaison de la fréquence des différents types histologiques selon certaines séries

	ADK bien différencié	ADK moyenne différencié	ADK peu différencié
Hajer Abaza	76%	17%	7%
Adolf	62,5%	22,5%	15%
Hamed Abdelouahab	50,9%	19,8%	4,6%
Pocard	39%	47%	14%
Notre série	58,7%	28%	5,3%

## Conclusion

L'analyse de nos résultats montre un profil épidémiologique particulier qui se caractérise par un âge plus jeune. Les carcinomes sporadiques sont largement prédominants avec une répartition topographique recto sigmoïdienne fréquemment observée. Sur le plan pronostique, le faible degré de différenciation des adénocarcinomes et le type mucineux sont corrélés à un stade TNM et d'Astler Coller avancés, avec un statut ganglionnaire péjoratif. Dans le monde entier le cancer colorectal suscite des inquiétudes par sa progression. Le Maroc n'échappe malheureusement pas à cette tendance.

### Etat des connaissances actuelle sur le sujet

- Cancer colorectal est très fréquent dans le monde;
- Il touche le plus souvent les sujets âgés de plus de 60 ans.

## Contribution de notre étude à la connaissance

- Un profil épidémiologique particulier au Maghreb qui se caractérise par un âge plus jeune, et une légère prédominance masculine;
- Les carcinomes sporadiques sont largement prédominants.

## Conflits d’intérêts

Les auteurs ne déclarent aucun conflit d'intérêts.
